# Sexual dimorphism in reactive oxygen species production and a role for integrin α1β1 in estrogen receptor α and β expression in articular cartilage

**DOI:** 10.1186/s13018-023-03655-2

**Published:** 2023-03-06

**Authors:** Alicia L. Black, James Haskins, Ambra Pozzi, Andrea L. Clark

**Affiliations:** 1 Department of Human Health and Nutritional Sciences, College of Biological Science, University of Guelph, 50 Stone Road East, Guelph, ON N1G 2W1 Canada; 2 Department of Medicine, Division of Nephrology and Hypertension, Vanderbilt University Medical Center, Nashville, TN USA; 3 Department of Veterans Affairs, Nashville, TN USA

**Keywords:** Integrins, Epidermal growth factor receptor, Estrogen receptor alpha, Estrogen receptor beta, Chondrocyte, Articular cartilage, Mice, Osteoarthritis

## Abstract

**Background:**

Osteoarthritis (OA) is a debilitating disease involving cartilage degradation. A need remains for the discovery of new molecular targets in cartilage for pharmaceutical intervention of OA. One potential target is integrin α1β1 that protects against OA when it is upregulated by chondrocytes early in the disease process. Integrin α1β1 offers this protection by dampening epidermal growth factor receptor (EGFR) signaling, and its effects are more robust in females compared to males. The aim of this study, therefore, was to measure the impact of *itga1* on chondrocyte EGFR activity and downstream reactive oxygen species (ROS) production in male and female mice. Furthermore, chondrocyte expression of estrogen receptor (ER) α and ERβ was measured to investigate the mechanism for sexual dimorphism in the EGFR/integrin α1β1 signaling axis. We hypothesized that integrin α1β1 would decrease ROS production and pEGFR and 3-nitrotyrosine expression, with this effect being greater in females. We further hypothesized that chondrocyte expression of ERα and ERβ would be greater in females compared to males, with a greater effect seen in *itga1*-null compared to wild-type mice.

**Materials and methods:**

Femoral and tibial cartilage of male and female, wild-type and *itga1*-null mice were processed for ex vivo confocal imaging of ROS, immunohistochemical analysis of 3-nitrotyrosine, or immunofluorescence of pEGFR and ERα and ERβ.

**Results:**

We show that ROS-producing chondrocytes are more abundant in female *itga1*-null compared to wild-type mice ex vivo*;* however, *itga1* had limited influence on the percent of chondrocytes stained positively for 3-nitrotyrosine or pEGFR in situ. In addition, we found that *itga1* influenced ERα and ERβ expression in femoral cartilage from female mice, and that ERα and ERβ were coexpressed as well as colocalized in chondrocytes. Finally, we show sexual dimorphism in ROS and 3-nitrotyrosine production, but surprisingly not in pEGFR expression.

**Conclusions:**

Together these data highlight sexual dimorphism in the EGFR/integrin α1β1 signaling axis and underline the need for further investigation into the role of ERs in this biological paradigm. Understanding the molecular mechanisms underlying the development of OA is essential for the development of individualized, sex-specific treatments in this age of personalized medicine.

## Introduction

Osteoarthritis (OA) is a degenerative joint disease characterized by articular cartilage degradation, meniscal damage, synovial inflammation and osteophyte formation at the joint margins [1]. For the patient, this manifests as joint pain and stiffness, and reduced mobility and quality of life. As such, OA is a leading cause of disability in North America affecting 1 in 6 adults [2, 3]. Many risk factors have been associated with OA including obesity, age, sex, and joint trauma, though the exact etiology of OA remains unknown [2]. Medication and therapy alleviate joint pain and inflammation and/or help to maintain or restore joint function; however, treatment options to slow or stop the progression of the disease itself remain elusive [1, 2]. Understanding the responses of chondrocytes to the early stages of OA may reveal novel molecular targets for treatment strategies that might delay the remodeling of the extracellular matrix, fibrillation, fissuring and ultimate destruction of the articular cartilage in joints.

Integrins are heterodimeric transmembrane extracellular matrix receptors and mediators of cell–cell interactions involved in the activation of numerous intracellular signaling cascades including regulation of cell adhesion, differentiation, and matrix remodeling [4, 5]. Of particular interest to OA is integrin α1β1 that is expressed by chondrocytes and binds collagens type II, IV, and VI and laminin in cartilage [6, 7]. Importantly, the α1 subunit pairs exclusively with the β1 subunit, and therefore, deletion of the α1 subunit in mice (*itga1*-null) renders them integrin α1β1 deficient, while maintaining the other partnerships of the β1 subunit [8]. Integrin α1β1 is upregulated in both subclinical and end-stage OA in cynomolgus macaque and mice [6, 9]. *Itga1*-null mice develop spontaneous OA three months earlier than wild-type mice [9] and post-traumatic OA one month earlier than wild-type mice, with the latter result exclusive to females [10]. Together these data suggest that integrin α1β1 protects against OA when it is upregulated in the early stages of disease, with a more robust influence in females compared to males. The molecular mechanisms by which integrin α1β1 affords this chondroprotection, however, are not well understood.

One possible mechanism for the protection of integrin α1β1 against OA is through its suppression of epidermal growth factor receptor (EGFR) signaling. Integrin α1β1 can control EGFR through T-cell protein tyrosine phosphatase (TCPTP) that can either dephosphorylate EGFR or act on caveolin-1, a scaffolding protein involved in EGFR activation [11]. Non-cartilaginous tissues in the *itga1*-null mouse have increased basal levels of phosphorylated EGFR and associated reactive oxygen species (ROS) production, increased levels of phosphorylated caveolin-1, and increased NADPH oxidase activity [11–15]. As such, one might expect EGFR activity to be upregulated in the chondrocytes of *itga1*-null mice, leading to increased catabolic activity in cartilage contributing to an earlier onset of OA. In this regard, we have shown that treatment with an EGFR inhibitor protected against cartilage degradation, with this effect being more robust in *itga1*-null compared to wild-type mice, and in female compared to male mice [10].

The research outlined above suggests that both integrin α1β1 and EGFR signaling have sex-dependent effects on cartilage. In a variety of tissues including articular cartilage, growth factors such as EGFR can activate estrogen receptor (ER) α and ERβ directly and indirectly through MAP or PI3/Akt kinase-mediated phosphorylation, in the absence of ER ligands [16, 17]. Therefore, changes in EGFR activity, mediated by integrin α1β1, could influence the expression of ERα and ERβ and contribute to the sexual dimorphism in knee OA in *itga1*-null mice.

Both ERα and ERβ have four functional domains in common including an amino-terminal A/B domain for gene transcription transactivation, a DNA binding C domain for ER dimerization, a hinge D region that allows the receptor-ligand complex to translocate to the nucleus, and finally a C-terminal E/F region containing the ligand binding domain for the binding of estrogen and other coactivators and corepressors [16, 17]. The main structural difference between ERα and ERβ is that ERβ has the shorter amino-terminal domain [16, 17]. Both ERα and ERβ have been identified in the articular cartilage of humans and various animals [18–20], with some studies reporting increased protein expression in females compared to males [21], while others report the opposite [22]. ER signaling is complex but can be summarized by three major pathways. Briefly, direct genomic signaling occurs where ligand-activated ERα and ERβ bind estrogen response elements in the promotor, or other transcription factors, to mediate transcription of genes [16, 17]. Second, non-genomic signaling occurs when ERα, ERβ or G protein-coupled receptor 30 (GPR30) at the cell membrane activate the MAP and/or PI3/Akt kinase-mediated signal transduction pathways [16, 17]. Finally, and of particular importance to this study, ERα and ERβ without ligands can be stimulated by growth factor receptors (including EGFR) either directly or via MAP or PI3K/Akt kinase-mediated phosphorylation [16, 17].

Therefore, the purpose of this study was to measure the impact of *itga1* on EGFR activity and downstream ROS production in mouse cartilage. In addition, chondrocyte expression of ERα and ERβ was measured to investigate the mechanism for sexual dimorphism in the EGFR/integrin α1β1 signaling axis. We hypothesized that there would be increased ROS production as well as increased expression of pEGFR and 3-nitrotyrosine in chondrocytes of *itga1*-null mice compared to wild-type controls, and that this effect would be greater in females compared to males. Finally, we also expected increased expression of ERα and ERβ in females compared to males, with a greater effect seen in *itga1*-null compared to wild-type mice.

## Materials and methods

### Animals

All methods were approved by the University of Guelph Animal Care Committee (AUP#3655). Skeletally mature (18 ± 2 weeks) wild-type and *itga1*-null [8], female and male BALB/c mice (*n* = 3 per group) were selected for experiments from breeding colonies at the University of Guelph. Genotype was determined through a multiplex polymerase chain reaction using DNA extracted from ear notches. Mice were weighed [mean ± standard deviation; wild-type female (28.1 ± 1.2 g) and male (31.1 ± 2.1 g), *itga1*-null female (27.4 ± 1.6 g) and male (33.0 g ± 2.2 g)] then anesthetized with isoflurane and euthanized by cardiac puncture followed by cervical dislocation [23]. Estrous cycle stage at euthanasia was not controlled.

### Ex vivo* ROS measurement in intact femora*

Left and right femora were dissected from male and female, wild-type and *itga1*-null BALB/c mice. Femora were kept hydrated with phosphate-buffered saline during dissection, before being submerged in iso-osmotic (300 mOsm), phenol red free, high glucose media (Thermo Fisher Scientific). Femora were incubated (37 °C and 5% CO_2_) with the live cell stain calcein AM (4 μM, Thermo Fisher Scientific) followed by dihydroethidium (10 μM, Calbiochem) which reacts with a variety of ROS to form 2-hydroxyethidium [24, 25].

For imaging, femora were placed condyles up in a 10-mm petri dish and submerged in media, maintained at 37 °C by a heat ring (Warner Instruments). Z-stack images of femoral condyles were generated using a 63 × /0.9 N.A. water immersion lens on a Leica DM 6000B confocal microscope connected to a Leica TCS SP5 scanner system (Leica Microsystems). Both stains were excited using a 488 nm laser. Calcein fluorescence was captured at 505–525 nm and 2-hydroxyethidium fluorescence at 615–635 nm. These collection bands are typically used for live/dead assays and pilot tests staining with calcein or dihydroethidium alone confirmed that the emission bands were far enough apart to stop bleed through of fluorescence between the channels. Z-stacks (40 μm) had a resolution of 1024 × 1024 pixels and a step size of 0.67 µm with the pinhole set to 0.5 Airy units (74.25 μm). To mitigate the effects of time post-staining on condylar differences in 2-hydroxyethidium fluorescence, the order of imaging was alternated between medial or lateral condyle.

### Cartilage preparation for immunohistochemical analysis

Femora and tibiae were isolated using micro-dissection, fixed overnight in 4% paraformaldehyde, decalcified for 32 h (Cal-Ex II, Thermo Fisher Scientific) and dehydrated (25% sucrose) overnight at 4 °C. Femora were trimmed to two thirds their length and tibiae were cut midshaft before being embedded in cryomolds (15 mm × 15 mm × 5 mm, VWR) filled with optimal cutting temperature medium (Thermo Fisher Scientific). In order to achieve physiological orientation of the femur, the shaft was balanced against the edge of the mold while the distal end was positioned in the center of the mold with the condyles facing up. Tibiae were oriented flat in the mold with the anterior side facing up. Cryosections (10 µm) were cut in the coronal plane (CM 3050S, Leica Microsystems) with four sections placed on each microscope slide (Superfrost Plus, Thermo Fisher Scientific) and stored at − 80 °C until use. A single slide from each of the three animals in each group was selected for immunohistochemistry or immunofluorescence, with all slides representing the center of the joint, within the cartilage–cartilage contact region of the medial and lateral femoral condyles/tibial plateaus. Section quality (tissue flat to the slide, no folds, or tears) was assessed using a brightfield microscope (Nikon Eclipse e400). Sections were then allocated to a treatment group (full antibody application, secondary only control, or blank control).

### Immunohistochemistry for 3-nitrotyrosine

Endogenous peroxidases were quenched (3% hydrogen peroxide in methanol) followed by blocking (4% normal horse serum) for one hour in a humidified chamber. Sections were then incubated with mouse monoclonal anti-nitrotyrosine antibody (1:500, ab61392, Abcam) overnight at 4 °C. The Ultra-Sensitive ABC Peroxidase Mouse IgG Staining Kit (Thermo Fisher Scientific) was used for the application of the secondary antibody as well as the ABC complex. Sections were developed with 3–3′-diaminobenzidine (Sigma-Aldrich) and counterstained with hematoxylin for imaging using light microscopy. Images were captured using a Nikon Eclipse e400 microscope outfitted with a Nikon Coolpix 990 digital camera. Using the 40 × /0.65 N.A. air objective lens, sequential images of the tissue sections were taken to capture the entire length and depth of the articular cartilage across both the medial and lateral femoral condyles/tibial plateaus.

### Immunofluorescence for ERα, ERβ and pEGFR

Sections were blocked (5% normal goat serum) for one hour at room temperature. To determine the presence of ERα and ERβ, anti-ERα mouse monoclonal (1:200, MA5-13191) and anti-ERβ rabbit polyclonal (1:200, PA1-310B) primary antibodies (Thermo Fisher Scientific) were diluted and applied simultaneously to sections to incubate overnight at 4 °C. Goat anti-mouse IgG (H + L) F(ab’)_2_ fragment Alexa Fluor 647 (1:500, A21237) and goat anti-rabbit IgG (H + L) Alexa Fluor 555 (1:500, A21428) secondary antibodies (Thermo Fisher Scientific) were then diluted and applied simultaneously to sections to incubate at room temperature for an hour. To determine the presence of pEGFR, anti-EGFR (phosphor 1092) rabbit monoclonal primary antibody (1:100, ab40815, Abcam) and goat anti-rabbit IgG (H + L) Alexa Fluor 555 secondary antibody (1:500, A21428, Thermo Fisher Scientific) were applied as described above. All sections were counterstained with 0.1 M Hoechst 33,342 (Invitrogen), coverslips applied (ProLong Gold, Thermo Fisher Scientific) and sealed.

Using a 40 × /0.75–1.25 N.A. oil objective (Leica DM 6000B confocal microscope with a TCS SP5 scanner system), a single confocal image was taken in the central cartilage–cartilage contact region of each compartment (medial/lateral) and site (femoral condyle/tibial plateau). Image resolution was set to 2048 × 2048 pixels with a two-line average and an optical slice thickness of 7.419 μm. A brightfield image and sequential fluorescent scans were captured to avoid interference between the florescent dyes. The following excitation and emission settings were used for each dye; ERα (ex 633 nm, em 670–720 nm), ERβ and pEGFR (ex 543 nm, em 570–600 nm or 625–640, respectively) and Hoechst (ex 405 nm, em 440–460 nm).

### Image analysis

**Fig. 1:**
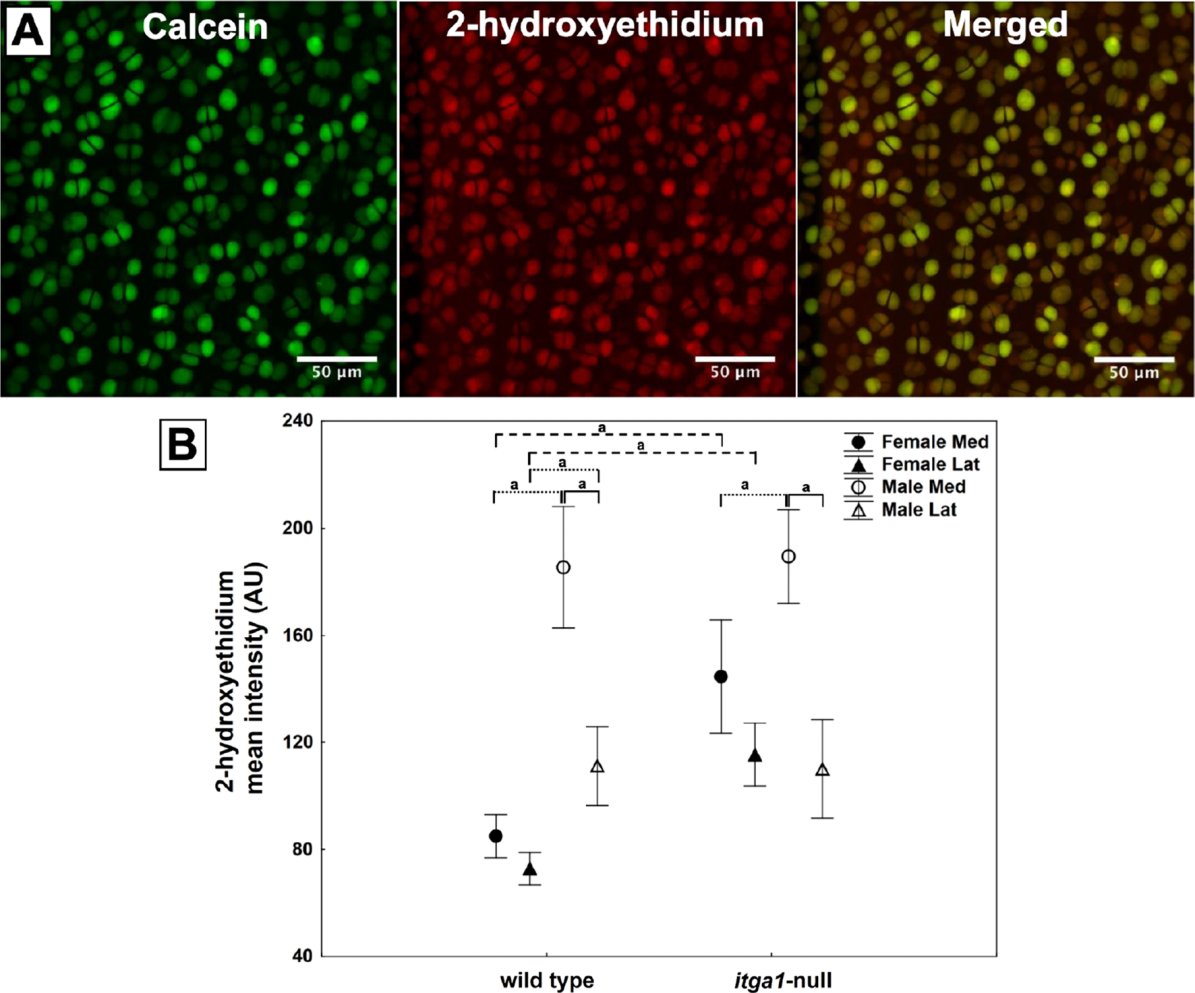
Representative 2-D projections of summed fluorescence throughout a Z-stack of ex vivo chondrocytes stained with calcein (live cell dye) and dihydroethidium (that reacts with a variety of ROS to form 2-hydroxyethidium) and the two images merged (**A**). Images taken from a medial femoral condyle of a male wild-type mouse. Mean intensity (arbitrary units) of 2-hydroxyethidium in femoral chondrocytes from the medial and lateral condyles of male and female wild-type and *itga1*-null mice (**B**). Data points are means (*N* = 3 femora, *n* = 90 cells) ± 95% CI. Genotype effect dashed bracket, sex effect dotted bracket and compartment effect solid bracket, *p* < 0.001 (a)

For the ex vivo ROS experiment, 2-D Z-projections of each condylar Z-stack were made using FIJI software (Fig. 1A). Cells were manually categorized as either live (calcein present throughout), or dead (calcein absent or speckled, indicating cell lysis) and the viability of the chondrocytes on each femoral condyle was calculated. Only condyles that had > 84% viability were analyzed. To measure 2-hydroxyethidium fluorescence, 2-D Z-projections of 30 individual live cells per condyle were generated from the original Z-stacks of the condyle. For each 2-D Z-projection of an individual chondrocyte, background fluorescence was calculated as the average of the extracellular fluorescence and subtracted from the entire 2-D Z-projection prior to calculating the mean intensity of 2-hydroxyethidium fluorescence.

For 3-nitrotyrosine immunohistochemistry, images were merged to create a collage of each femoral condyle/tibial plateau and the tidemark was marked using the ‘draw’ tool (PowerPoint). Sample collages were randomized and the total number of chondrocytes in the articular cartilage, and those stained positively for 3-nitrotyrosine were manually quantified by two blinded graders. Zonal analysis across the depth of the cartilage was not conducted as the zones could not be distinguished in the thin femoral cartilage (20–30 µm, 3–4 cell layers). For pEGFR, ERα and ERβ immunofluorescence, the tide mark and articular surface of the cartilage seen on the differential interference contrast images were marked (ImageJ), and the images randomized ready for counting. The total number of chondrocytes and those stained positively for pEGFR, ERα alone, ERβ alone or both ERα and ERβ were manually counted by two blinded graders and tallied electronically (ImageJ).

### Statistical analysis

For the immunohistochemical and immunofluorescence analyses, Pearson correlations were run between the data sets from the two graders. Pearson correlation coefficients of 0.88 for 3-nitrotyrosine counts, 0.97 for pEGFR counts, and 0.97 for ERα and ERβ counts were obtained and therefore the cell counts of the two graders were averaged and percent positive cells was calculated [26]. The mean 2-hydroxyethidium fluorescence measurements as well as the percent of chondrocytes positively stained for 3-nitrotyrosine or pEGFR were subject to an ANOVA, while the ER data were subject to a repeated measures ANOVA, all with sex (male/female), genotype (wild-type/*itga1*-null), site (femoral condyle/tibial plateau) and condyle (medial/lateral) as factors (Statistica™). Fisher LSD post hoc tests were conducted where appropriate and significance was set at *p* < 0.05.

## Results

### *Itga1-null, sex and compartment affect ROS activity *ex vivo

2-hydroxyethidium is an ex vivo fluorescent product of a direct reaction between a variety of ROS and the small molecule dihydroethidium which can be analyzed as a measure of ROS activity in live cells [24, 25]. On average, basal levels of 2-hydroxyethidium were larger in chondrocytes from male compared to female mice with this effect being more pronounced on the medial compared to the lateral condyle (*p* < 0.04) (Fig. 1B). Furthermore, 2-hydroxyethidium was unaffected by genotype in male mice but was greater in female *itga1*-null compared to female wild-type mice (*p* < 0.001) (Fig. 1B).

### *Sex affects 3-nitrotyrosine expression *in situ

**Fig. 2:**
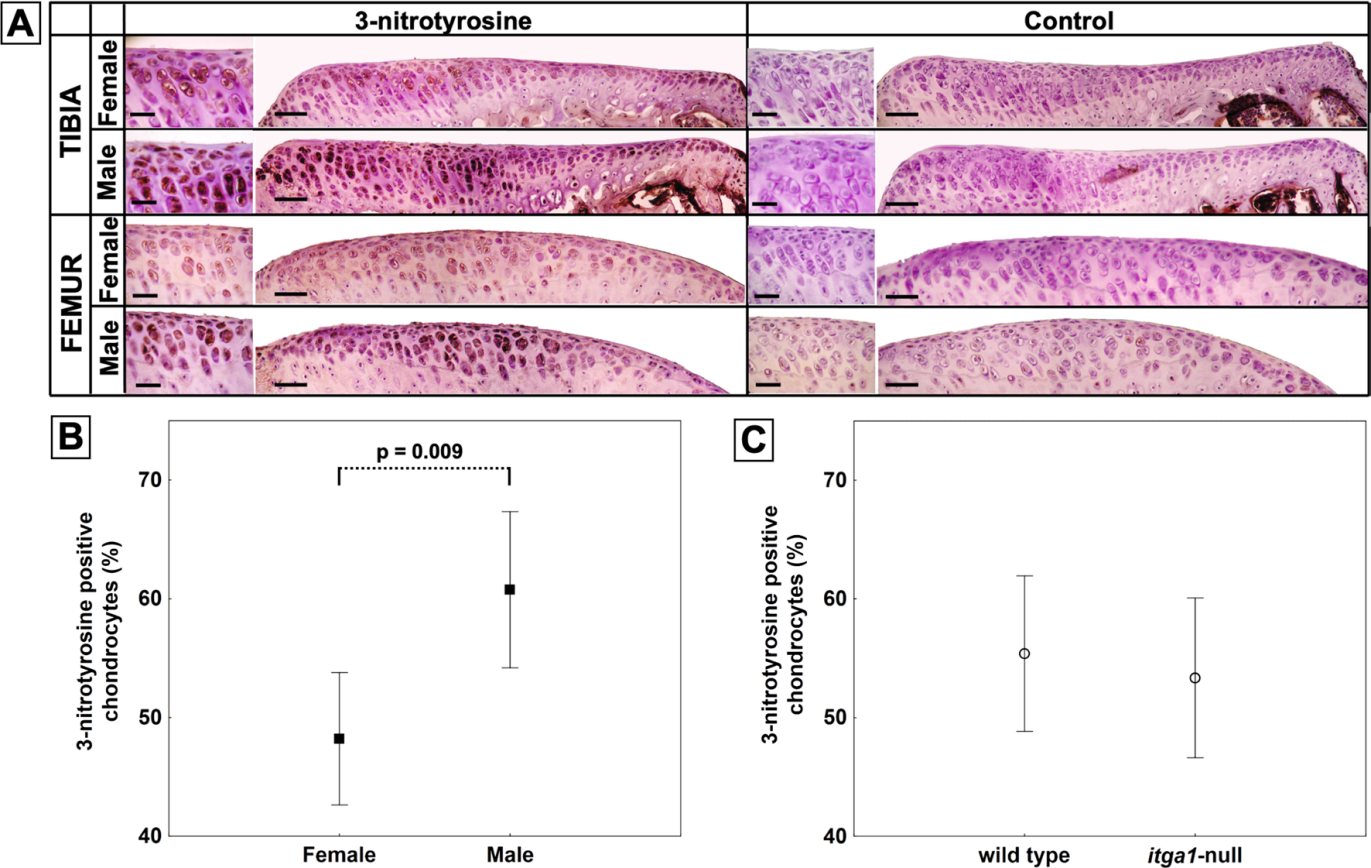
Light microscopy images of the full thickness articular cartilage of the medial tibial plateau or medial femoral condyle from female or male, wild-type (tibia) or *itga1*-null (femur), mice (**A**). Sections are peroxidase stained for 3-nitrotyrosine or secondary only control. Scale bar 20 µm or 50 µm in high- or low-magnification images, respectively. Note the increased staining in tissues from male compared to female mice. Percent of chondrocytes stained positively for 3-nitrotyrosine as a function of sex (**B**) or genotype (**C**). Data points are means (*N* = 6 femora, *n* > 1000 cells) ± 95% CI

3-nitrotyrosine is an in situ indirect measure of ROS (the byproduct of superoxide reacting with nitric oxide and then with proteins [27]). Chondrocytes stained positively for 3-nitrotyrosine throughout cartilage depth across the tibial plateaus and femoral condyles relative to secondary only control sections (Fig. 2A). On average, males had 10% more chondrocytes stained positively for 3-nitrotyrosine compared to females (*p* = 0.009) (Fig. 2B). Genotype, site, and compartment did not affect 3-nitrotyrosine staining [(*p* > 0.05) Fig. 2C or data not shown].

### *Itga1-null and site affect pEGFR expression *in situ

**Fig. 3:**
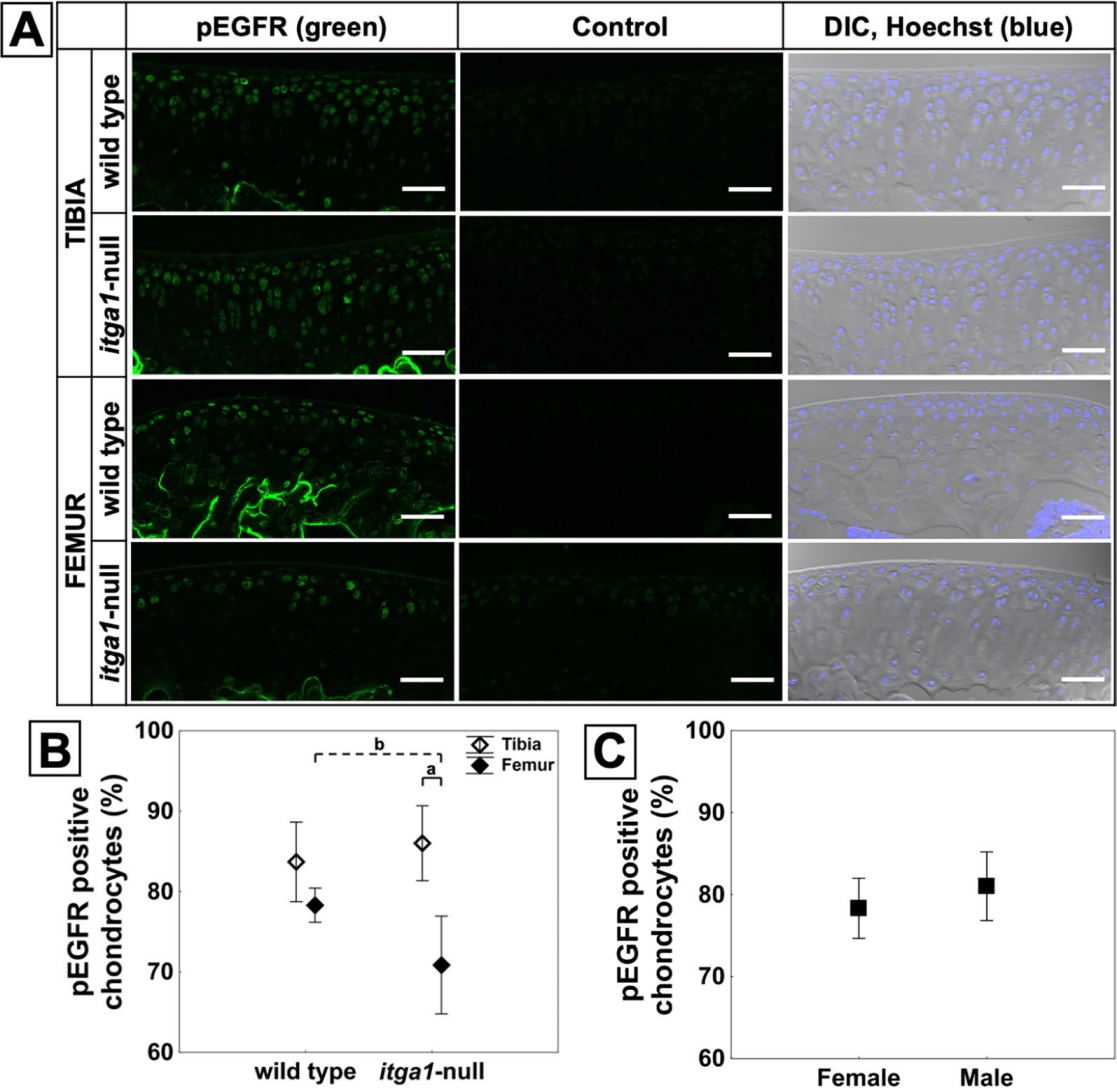
Confocal microscopy images of tibial and femoral cryosections showing the articular cartilage from the lateral compartment of *itga1*-null and wild-type male mice immunostained green for pEGFR (**A**). Secondary antibody only control sections show negligible autofluorescence from the murine cartilage. Nuclei were counterstained blue with Hoechst as seen in the differential interference contrast (DIC) images. Scale bar 50 μm. Percent of chondrocytes stained positively for pEGFR as a function of genotype and site (**B**) or sex (**C**). Data points are means (*N* = 6 femora, *n* > 800 cells) ± 95% CI. Genotype effect dashed bracket and site effect solid bracket, *p* < 0.001 (a), *p* = 0.014 (b)

Chondrocytes stained positively for pEGFR throughout cartilage depth across the tibial plateaus and femoral condyles relative to secondary only control sections (Fig. 3A). Chondrocyte expression of pEGFR was affected by a genotype and site interaction (*p* = 0.014) (Fig. 3B). Specifically, 15% more *itga1*-null chondrocytes stained positively for pEGFR on the tibial plateaus compared to the femoral condyles (*p* < 0.001), but there was no site effect in wild-type mice. Furthermore, 8% more femoral chondrocytes stained positively for pEGFR in wild-type compared to *itga1*-null mice (*p* = 0.014); however, there was no genotype effect in the tibia. Sex and compartment had no influence upon chondrocyte expression of pEGFR [(*p* > 0.05) Fig. 3C or data not shown].

### *Itga1-null, sex and site affect ERα and ERβ expression *in situ

**Fig. 4:**
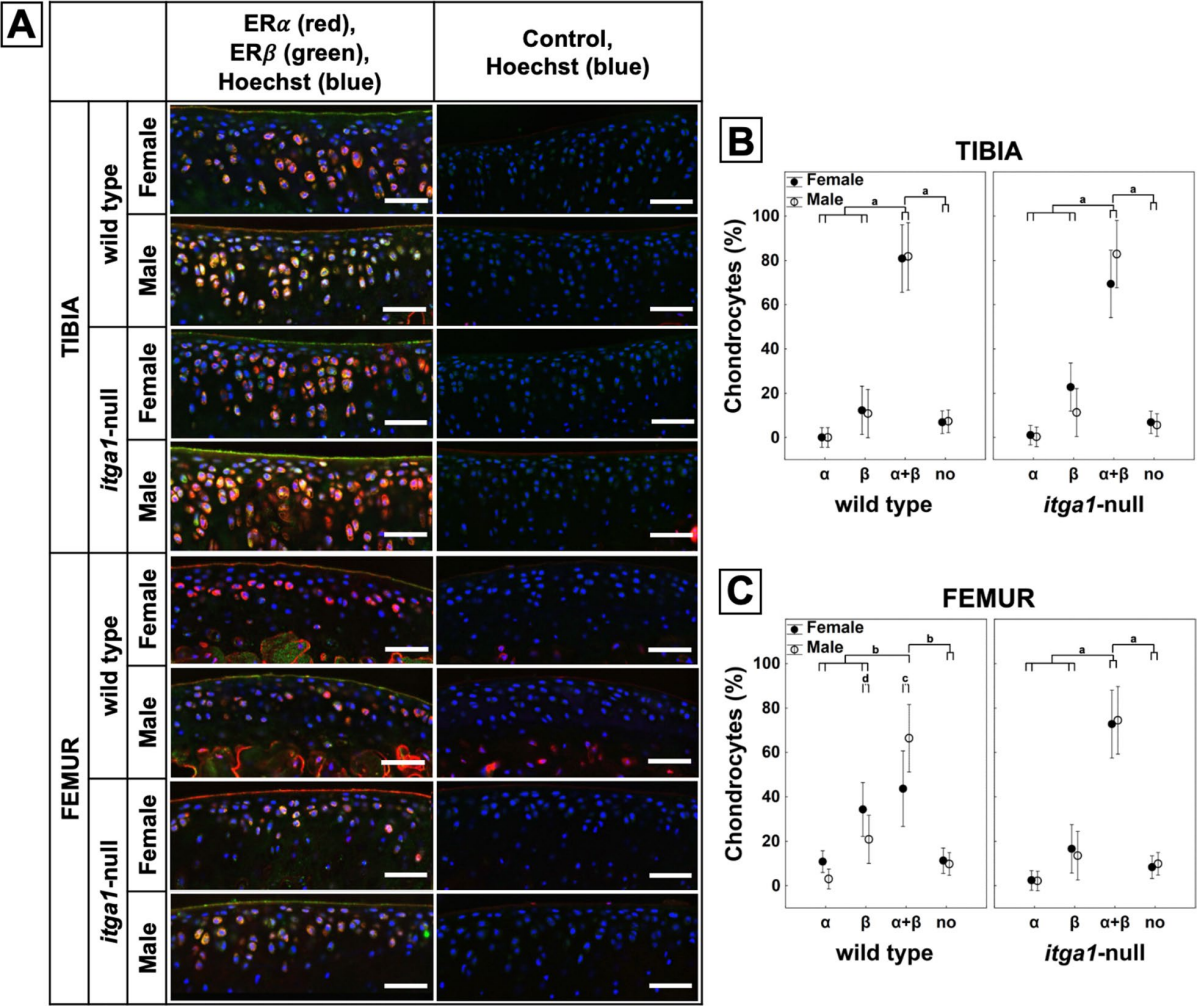
Confocal microscopy images of full thickness articular cartilage from the lateral tibial plateau and the medial femoral condyle of female and male *itga1*-null and wild-type mice (**A**). Chondrocytes stained red for ER⍺, green for ERβ or secondary only controls. All sections counterstained blue with Hoechst. Scale bar 50 µm. Percent of tibial (**B**) and femoral (**C**) chondrocytes stained positively for ER⍺, ERβ, ER⍺ + β, or no receptor as a function of genotype and sex. Note the expression of ER⍺ + β is significantly greater than either receptor alone or no receptor, except in femoral chondrocytes from wild-type females. Data points are means (*N* = 3 femora, *n* > 400 cells) ± 95% CI. *p* < 0.001 (a), *p* < 0.006 (b), *p* = 0.002 (c), *p* = 0.04 (d)

**Fig. 5:**
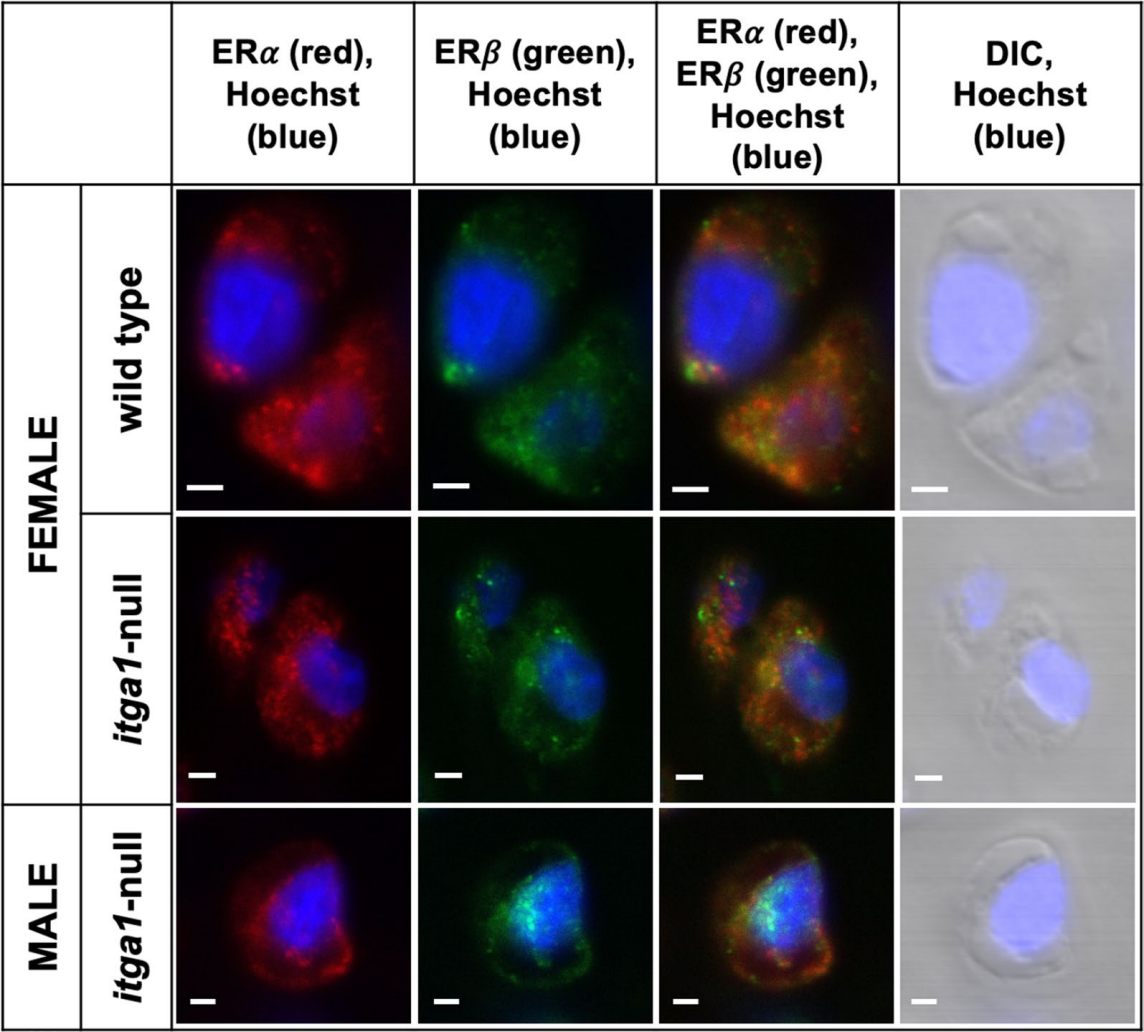
Digitally zoomed confocal microscopy images of chondrocytes from the lateral tibial plateaus of a female wild-type or *itga1*-null mice, and a male *itga1*-null mice. Images show ERα stained red, ERβ stained green, overlay of ERα and ERβ, or differential interference contrast (DIC). All nuclei counterstained blue with Hoechst. Note the presence of ERα and ERβ in the cytoplasm and/or nucleus, and colocalization of ERα and ERβ in the cytoplasm. Scale bar 2 μm

Chondrocytes stained positively for ER⍺ and ERβ throughout cartilage depth across the tibial plateaus and femoral condyles relative to secondary only control sections (Fig. 4A). ER⍺ and ERβ were present in the nucleus as well as the cytoplasm of chondrocytes and the receptors were sometimes colocalized in the cytoplasm (Fig. 5). The percent of chondrocytes stained positively for ERs was influenced by sex (*p* = 0.028) as well as a genotype and site interaction (*p* = 0.004). In the tibia (Fig. 4B), the percent of chondrocytes stained positively for both ER⍺ and ERβ was at least 40% greater than either receptor alone or no receptor, independent of genotype and sex (*p* < 0.001). This expression pattern was also observed in femoral chondrocytes (Fig. 4C) from male and female *itga1*-null mice (*p* < 0.001) and male wild-type mice (*p* < 0.006). In femoral chondrocytes from female wild-type mice, however, similar numbers (40%) of chondrocytes were stained positively for either ERβ alone or both ER⍺ and ERβ.

## Discussion

The purpose of this study was to measure the impact of *itga1* on EGFR activity and downstream ROS production in mouse cartilage. In addition, chondrocyte expression of ERα and ERβ was measured as a potential mechanism for sexual dimorphism in the EGFR/integrin α1β1 signaling axis. We show that ROS producing chondrocytes are more abundant in female *itga1*-null compared to female wild-type mice ex vivo*;* however, *itga1* had limited influence on the percent of chondrocytes stained positively for 3-nitrotyrosine or pEGFR in situ. In addition, we found that *itga1* influenced ERα and ERβ expression in femoral cartilage from female mice, and that ERα and ERβ were coexpressed as well as colocalized in chondrocytes. Finally, we show sexual dimorphism in ROS and 3-nitrotyrosine production, but surprisingly not in pEGFR expression.

Firstly, in agreement with our hypothesis we show that *itga1*-null chondrocytes have more ROS production compared to their wild-type counterparts when measured ex vivo in murine femora, but only in female mice. This is in agreement with previous work from our lab that saw earlier cartilage damage in female *itga1*-null compared to wild-type mice following surgery to destabilize the medial meniscus, which was ameliorated with the EGFR antagonist erlotinib [10]. Together, these studies provide evidence to support the protective role of integrin α1β1 in cartilage through suppression of EGFR activity.

In contrast to our hypothesis, however, we show that *itga1* had limited influence on the percent of chondrocytes stained positively for 3-nitrotyrosine and pEGFR in situ. The divergence in these results may be reflective of our use of a subclinical as opposed to a surgical intervention model of OA in mice, and the contrasting measures of ROS production used. The expression of integrin α1β1 in cartilage increases significantly during matrix remodeling associated with early OA [9]. Such remodeling was not apparent in the 4-month-old mice used in our experiments. In terms of our measures of ROS production, 3-nitrotyrosine is an in situ indirect measure of ROS (the byproduct of superoxide reacting with nitric oxide and then with proteins [27]) whereas 2-hydroxyethidium is an ex vivo fluorescent product of a direct reaction between a variety of ROS and the small molecule dihydroethidium [24, 25]. Thus, the dampened expression of integrin α1β1, and the indirect measure of ROS used, may have hampered our ability to demonstrate a genotypic effect in pEGFR and ROS in situ.

We also found that *itga1* influenced ERα and ERβ expression in cartilage from female mice. Specifically, 20% more chondrocytes stained positively for both ERα and ERβ in cartilage from female *itga1*-null femora and this was compensated for by 20% fewer chondrocytes stained positively for ERβ alone, when compared to wild-type mice. In parallel to our result, it has been shown that cells induced to express ERβ increased integrin α1β1 mRNA and protein content as well as increased adhesion to extracellular matrix components such as laminin in breast cancer cell culture [28]. Interestingly, however, the in vitro treatment of endometrial cells with estrogen had no effect on the expression of various integrins, including integrin α1β1 [29]. These studies together with our findings suggest that integrin α1β1 and ERs can influence the expression and activation of each other independent of estrogen and thus may contribute to the sexual dimorphism seen in the effect of integrin α1β1 on the development of post-traumatic OA in mice [10].

Independent of the effects of *itga1*, we observed that the vast majority (70%) of chondrocytes express both ER⍺ and ERβ which are often colocalized and are present at the cell surface, in the cytoplasm, and nucleus. Our data are consistent with other reports of ER isoform distribution in various models including rats and cultured human chondrocytes [30–32]; however, we show their colocalization in chondrocytes for the first time. It is known that both ligand-dependent and -independent ER activity can influence chondrocyte biological activity, and that ER can translocate to the nucleus to influence gene expression in chondrocytes [16]. ER⍺ and ERβ form homodimers and/or heterodimers upon activation [33] and bind to the same DNA sequences (estrogen response elements) in vitro, thus jointly regulating DNA transcription in response to activation [34, 35]. Together with our work, this implies that through colocalization of the two dominant isoforms, ER⍺ and ERβ, the receptors may act cooperatively to elicit their cellular actions in chondrocytes.

Independent of *itga1*, we measured sexual dimorphism in chondrocyte ROS production, with male mice expressing more ROS than their female counterparts. Specifically, ROS levels ex vivo were at least 30% greater in chondrocytes from male compared to female mice and 10% more chondrocytes in situ stained positively for 3-nitrotyrosine in males compared to females. While additional research in chondrocytes is lacking, our findings agree with other studies in non-cartilaginous tissues that saw increased antioxidant enzyme activity in various organs in female compared to male mice [36], and increased ROS production in males compared to females in the analysis of human blood samples [37], and in microvessels of hypertensive rats [38]. In contrast to our ROS results and contrary to our hypothesis, sexual dimorphism was absent in our measurements of pEGFR. This is in contrast to our previous work that saw increased pEGFR expression in male compared to female mice 12 weeks following DMM surgery [10]. As the same antibody was used in both experiments, this disparity is likely due to surgery exacerbating the sexual dimorphism making it measurable in the post-traumatic OA model but not in our subclinical model. Taken together, this evidence suggests that activation of EGFR is stimulated by an injurious or traumatic event, potentially affecting chondrocyte ROS production and thus cartilage degradation in a sex-dependent manner.

While our work has focused on the dampening of EGFR signaling by integrin α1β1 and its potential to influence ERα and ERβ expression, it is important to note that ER signaling can also influence EGFR signaling. ERα and ERβ pooled and associated with the plasma membrane and/or estrogen bound GPR30 can promote EGFR activation [17, 39]. Future investigation into the extent of bidirectional ER and EGFR cross-talk in chondrocytes and its influence on osteoarthritis would be justified. In addition to ERs, androgen receptor expression has been confirmed in rat growth plate chondrocytes and rabbit articular chondrocytes of both sexes, and overexpression can protect articular chondrocytes from apoptosis [22, 40]. Furthermore, it has been shown that growth factor receptors, including EGFR, cross-talk with androgen receptor in prostate cancer and epithelial cells [41, 42]. In our *itga1*-null model therefore, it is possible that elevated EGFR signaling may influence androgen receptor activity in chondrocytes. This warrants further investigation.

## Conclusions

In conclusion, we show that ROS-producing chondrocytes are more abundant in female *itga1*-null compared to wild-type mice ex vivo*.* In addition, we found that *itga1* influenced ERα and ERβ expression in femoral cartilage from female mice, and that ERα and ERβ were coexpressed as well as colocalized in chondrocytes. Finally, we show sexual dimorphism in 3-nitrotyrosine production, but surprisingly not in pEGFR expression. Taken together, these data provide further support for sexual dimorphism in the EGFR/integrin α1β1 signaling axis and underline the need for further investigation into the role of ER in this biological paradigm.

## Data Availability

The datasets used and/or analyzed during the current study are available from the corresponding author on reasonable request.

## Abbreviations

EGFR: Epidermal growth factor receptor
pEGFR: Phosphorylated epidermal growth factor receptor
ER: Estrogen receptor
GPR30: G protein-coupled receptor 30
OA: Osteoarthritis
ROS: Reactive oxygen species
TCPTP: T cell protein tyrosine phosphatase
